# Supplementary material for: Novel Driver Strength Index highlights important cancer genes in TCGA PanCanAtlas patients
Source: PeerJ. 2022 Aug 11;10:e13860. doi: 10.7717/peerj.13860 (PMC9375969; doi:10.7717/peerj.13860)
Supplement: Supplemental Information 2 [file peerj-10-13860-s002.zip › COHORTS/genes plots/detailed/2021_8_16_14_9_distribution_events_detailed_males_29.pdf]

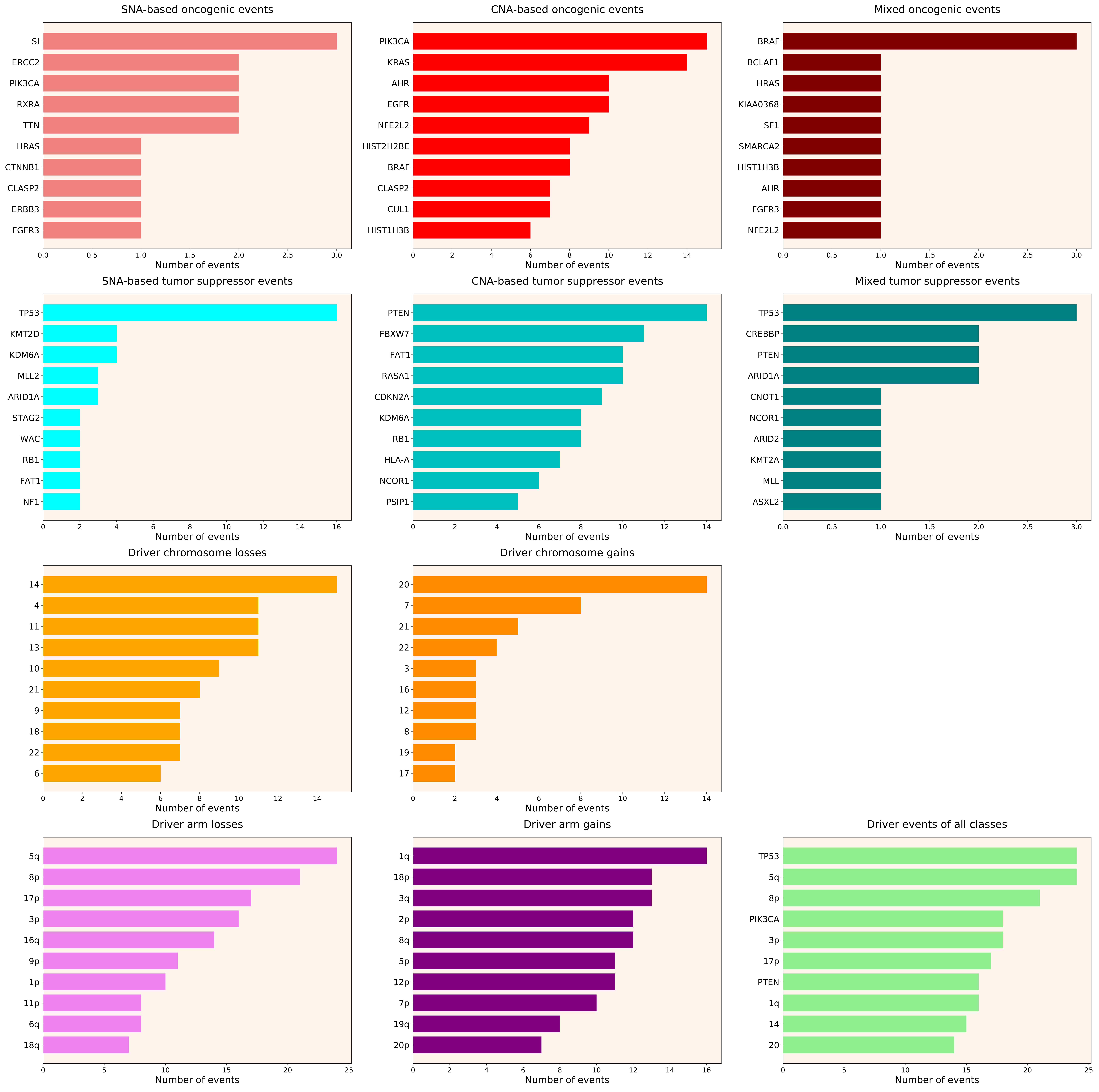
SNA-based oncogenic events
SI
ERCC2
PIK3CA
RXRA
TTN
HRAS
CTNNB1
CLASP2
ERBB3
FGFR3
Number of events
CNA-based oncogenic events
PIK3CA
KRAS
AHR
EGFR
NFE2L2
HIST2H2BE
BRAF
CLASP2
CUL1
HIST1H3B
Number of events
Mixed oncogenic events
BRAF
BCLAF1
HRAS
KIAA0368
SF1
SMARCA2
HIST1H3B
AHR
FGFR3
NFE2L2
Number of events
SNA-based tumor suppressor events
TP53
KMT2D
KDM6A
MLL2
ARID1A
STAG2
WAC
RB1
FAT1
NF1
Number of events
CNA-based tumor suppressor events
PTEN
FBXW7
FAT1
RASA1
CDKN2A
KDM6A
RB1
HLA-A
NCOR1
PSIP1
Number of events
Mixed tumor suppressor events
TP53
CREBBP
PTEN
ARID1A
CNOT1
NCOR1
ARID2
KMT2A
MLL
ASXL2
Number of events
Driver chromosome losses
14
4
11
13
10
21
9
18
22
6
Number of events
Driver chromosome gains
20
7
21
22
3
16
12
8
19
17
Number of events
Driver arm losses
5q
8p
17p
3p
16q
9p
1p
11p
6q
18q
Number of events
Driver arm gains
1q
18p
3q
2p
8q
5p
12p
7p
19q
20p
Number of events
Driver events of all classes
TP53
5q
8p
PIK3CA
3p
17p
PTEN
1q
14
20
Number of events
